# Properties Evaluation of a Novel Entropy-Stabilized Ceramic (La_0.25_Ce_0.25_Nd_0.25_Sm_0.25_)Ti_2_Al_9_O_19_ with Enhanced CMAS Corrosion Resistance for Thermal Barrier Coating Applications

**DOI:** 10.3390/ma18081778

**Published:** 2025-04-13

**Authors:** Fuxing Ye, Ziqi Song, Fanwei Meng, Sajid Ali

**Affiliations:** 1 Tianjin Key Laboratory of Advanced Joining Technology, School of Materials Science and Engineering, Tianjin University, Tianjin 300072, China; songziqi125@163.com (Z.S.); fwmeng@tju.edu.cn (F.M.); sajji2016@hotmail.com (S.A.); 2 Key Laboratory of Advanced Ceramics and Machining Technology of Ministry of Education, Tianjin 300072, China

**Keywords:** entropy-stabilized ceramic, thermal barrier coating, thermophysical property, CMAS corrosion

## Abstract

In this work, a novel potential thermal barrier coating material entropy-stabilized titanium–aluminum oxide (La_0.25_Ce_0.25_Nd_0.25_Sm_0.25_)Ti_2_Al_9_O_19_ (META) was successfully synthesized by the solid-state reaction method, and its thermophysical properties, phase stability, infrared emissivity, mechanical properties, and CMAS corrosion resistance were systematically investigated. The results demonstrated that META exhibits low thermal conductivity at 1100 °C (1.84 W·(m·K)^−1^), with a thermal expansion coefficient (10.50 × 10^−6^ K^−1^, 1000–1100 °C) comparable to yttria-stabilized zirconia (YSZ). Furthermore, META displayed desirable thermal stability, high emissivity within the wavelength range of 2.5–10 μm, and improved mechanical properties. Finally, META offers superior corrosion resistance due to its excellent infiltration inhibiting. The bi-layer structure on the corrosion surface prevents the penetration of the molten CMAS. Additionally, doping small-radius rare-earth elements thermodynamically stabilizes the reaction layer. The results of this study indicate that (La_0.25_Ce_0.25_Nd_0.25_Sm_0.25_)Ti_2_Al_9_O_19_ has the potential to be a promising candidate for thermal barrier coating materials.

## 1. Introduction

Thermal barrier coatings (TBCs) are widely utilized in gas turbine components to protect the alloy substrate from a high-temperature service environment, which can enhance fuel efficiency and improve the thrust-to-weight ratio of engines through the use of materials with low thermal conductivity [1,2,3,4]. Currently, the industry standard for the topcoat materials of TBCs is 6–8 wt.% Y_2_O_3_-stabilized zirconia (YSZ) [5]. As aerospace engines and gas turbines evolve towards higher operating temperatures, thrust-to-weight ratios, and flow ratios, the TBCs’ material is confronting new challenges [6]. In order to ensure an optimal performance in challenging operating environments, it is essential to optimize the mechanical, thermophysical, and chemical properties associated with the thermal barrier coating materials. This is particularly important in cases where adverse and complex operating environments are present, such as high velocity flame impingement and calcium–magnesium–aluminum–silicate (CMAS) erosion. In the context of the operation of an aero-engine, a range of materials, including environmental dust, debris, volcanic ash, runway debris, and impurities in the fuel, are drawn into the gas turbine. As a consequence, some of these particles melt and deposit on the surface of the gas turbine blades, forming a glassy layer of molten salt. The main components are mixed oxides of CaO, MgO, Al_2_O_3_, and SiO_2_, hence the name CMAS molten salt [7]. However, the ability to arrest CMAS corrosion of TBCs needs to be strengthened further. Furthermore, the corrosion failure process of CMAS infiltration into TBCs involves both physical and chemical processes. Firstly, the molten CMAS penetrates the pores of the TBCs by capillary action. During the subsequent cooling phase, the coefficient of thermal expansion (CTE) of CMAS undergoes a rapid change, resulting in severe damage of the TBCs’ structure due to thermal stress. Secondly, the molten glass will react with the TBCs, forming apatite and silicate, etc., significantly reducing strain tolerance and thermal cycle life. Meanwhile, YSZ materials undergo rapid volume changes with phase transitions during CMAS corrosion, resulting in a rapid failure rate. According to Planck’s law, at a temperature of 1000 °C, a blackbody in thermal equilibrium emits 97% of its electromagnetic energy below 14 μm, with 76% of the energy emitted below 5 μm [8]. Wien’s displacement law indicates that the peak wavelength of radiant emittance shifts to shorter wavelengths when the temperature increases [9]. Therefore, the development of near-infrared high emissivity materials is highly beneficial for applications requiring high temperatures. Conversely, YSZ exhibits low emissivity at wavelengths shorter than 5 μm [10]. In general, the necessity for the development of new TBCs that are capable of matching the performance of more demanding operating environments is of paramount importance.

To address these stringent requirements, various candidate TBC materials have been explored [11,12,13,14,15,16]. However, further enhancements are necessary to optimize the performance of these materials in terms of molten salt corrosion resistance, in order to better align them with the performance requirements of TBCs. Recently, lanthanum–titanium–aluminum oxide (LaTi_2_Al_9_O_19_, LTA) has attracted considerable interest due to its low density and thermal conductivity (2.4 W·(m·K)^−1^, 1100 °C) [17,18,19,20,21]. LTA has a CTE similar to that of YSZ and exhibits a superior thermal cycling performance when used as the top layer in a double-coating system with YSZ, making it a promising candidate for thermal protection applications [16]. Since Rost et al. introduced the concept of high-entropy ceramics in 2015 [22,23], high-entropy and mid-entropy ceramic materials have been recognized for their enhanced thermophysical properties and improved corrosion resistance, owing to the high-entropy effect, the lattice distortion effect, the cocktail effect, and the hysteresis diffusion effect. The entropy stabilization effect provides a new approach to improve the performance of TBCs’ material [24,25,26,27,28,29]. In addition, this combination of properties renders them particularly well-suited for utilization in TBCs.

In this study, a new type of mid-entropy titanium–aluminum oxide ceramic (La_0.25_Ce_0.25_Nd_0.25_Sm_0.25_)Ti_2_Al_9_O_19_ (META) was designed and successfully synthesized using the solid-state reaction method, for which the phase structure, microstructure, thermophysical and mechanical properties, infrared emissivity, and CMAS corrosion behavior were systematically investigated.

## 2. Experimental Procedures

### 2.1. Sample Preparation

The META ceramic samples were synthesized using the solid-state reaction method. The raw materials employed in the synthesis are commercially available La_2_O_3_, CeO_2_, Nd_2_O_3_, and Sm_2_O_3_ (≥99.9%, HWRK Chem Co., Ltd., Beijing, China), and Al_2_O_3_ and TiO_2_ powders (≥99.9%, Aladin Biochemical Co., Ltd., Shanghai, China). The powders were weighed in a stoichiometric ratio according to the target composition, and were ball-milled with zirconia balls in an anhydrous ethanol medium at 400 rpm for 8 h, maintaining a mass ratio of 1:2 for powders and balls. Subsequently, the slurry was subjected to a drying process at 100 °C for 12 h in a dried oven. Following the drying process, the powder was passed through a 200-mesh sieve after being ground. The sieved powder was uniaxially pressed into cylindrical pellets, using a stainless-steel die with a diameter of 15 mm and under a pressure of 250 MPa for 180 s. This process was undertaken in order to form green bodies, which will be sintered and then used for property and corrosion testing. Additionally, strip pellets (25 mm × 5 mm × 5 mm) were produced using the aforementioned method for thermal expansion testing after sintering. Ultimately, the green bodies underwent sintering at 1400 °C for 24 h in an air environment with a temperature rise rate of 5 °C·min^−1^, employing a muffle furnace and resulting in the formation of the META ceramic bulks.

### 2.2. CMAS Corrosion Tests

The CMAS powder was prepared using the melt-quenching method according to the molar ratio of 33CaO–9MgO–13AlO_1.5_–45SiO_2_. Then, the CMAS powder was evenly spread on the surface of META ceramic bulks at a density of 20 mg·cm^−2^ after the samples were cleaned with water and ultrasonic treatment. The CMAS-coated META ceramics were heated at 1250 °C for 3, 6, 12, and 24 h, and at 1300 °C for 6 and 24 h for a higher temperature comparison. The raw powder was heated at 1400 °C for 3 h with a heating rate of 5 °C·min^−1^ to synthesize the META ceramic powder, and the synthesized META ceramic powder was mixed with CMAS powder at a mass ratio of 10:3. The resulting mixture was uniaxially pressed into cylindrical pellets with a diameter of 15 mm, and was subjected to the same heating process as the corrosion experiment to further analyze the corrosion products.

### 2.3. Characterizations

The phase structure of the META ceramic samples was determined using X-ray diffraction (XRD, D8-ADVANCE, Cu Kα radiation, Bruker, Ettlingen, Germany). The surface morphology and corrosion interface of the sintered META ceramics, as well as the elemental distribution, were analyzed using a scanning electron microscope (SEM, HITACHI S-4800, Tokyo, Japan) equipped with energy dispersive spectrometry (EDS). The infrared emissivity of the META ceramics at 2.5–14 μm was measured using the FTIR Spectrometer (Nicolet IS50, Thermo Fisher Scientific, Waltham, MA, USA). Hardness and fracture toughness tests were conducted using a Vickers hardness tester with a 2 kg load applied for 15 s. The fracture toughness (K_IC_) was calculated using the indentation method with the following formula:

K_IC_ = δH_V_ a^2^ c ^(−3/2)^ (1)

where *δ*, *H_V_*, *a*, and *c* are the constant (0.16), Vickers hardness value, half-length of the indentation diagonal, and the distance from the indenter center to the tip of extended cracks, respectively. The CTE of the META ceramic was measured using a high-temperature dilatometer (NETZSCH DIL 402 C, Selb, Germany) from 30 °C to 1300 °C, with a heating rate of 5 °C·min^−1^ in an air environment. The CTE is calculated using the following equation:

α = (dL/L_0_)/∆T (2)

where *dL, L*_0_, and ∆*T* are the length change of the sample, the initial length of the sample at room temperature, and the temperature change, respectively. The thermal diffusivity (κ_0_) of META was measured from 300 °C to 1100 °C using the laser flash method (NETZSCH LFA 467, NETZSCH Analyzing & Testing, Selb, Germany) at an interval of 200 °C. The thermal diffusivity of complete dense (κ) is corrected for porosity using the following formula:

κ/κ_0_ = 1 − 4/3 Φ (3)

where the *Φ* is the porosity of the bulk sample, calculated as follows:

Φ = 1 − ρ/ρ_0_ (4)

where ρ_0_, ρ, are the calculated theoretical density and measured bulk sample density. The thermal conductivity (λ) of META was calculated using the following formula:

λ = C_P_ × ρ × κ (5)

where *C_P_* is the specific heat capacity of META, calculated using the Neumann–Kopp rule. The theoretical density ($\rho_{th}$) of META is calculated from following equation:

(6) $$\rho_{th}=\frac{\sum M}{V_{0}\times N}$$

where ∑M is the sum of the molar masses of all atoms contained within a unit lattice, $V_{0}$ is the unit lattice volume calculated from lattice constants, and N is the Avogadro’s constant.

## 3. Results and Discussion

### 3.1. Phase Composition and Microstructure Characterization

**Figure 1:**
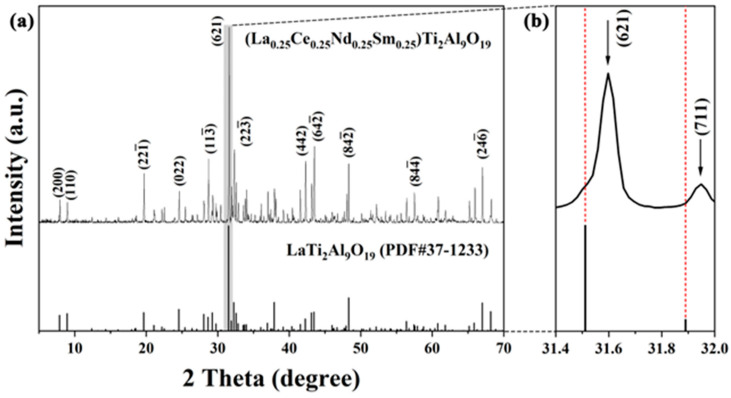
(**a**) XRD patterns of synthesized META ceramic bulk; (**b**) partial enlargement of the (621) peak at the 2θ positions of 31.4–32°.

Figure 1a illustrates the X-ray diffraction (XRD) patterns of the META ceramic bulk synthesized at 1400 °C using the solid-state reaction method. A comparison of the experimental XRD pattern with the standard PDF card reveals that the primary diffraction peaks of the sintered META ceramic align with those of single-component LTA. This confirms that META ceramics have structurally the same phase structure as pure LTA with no detectable secondary phases. A partial enlargement of the (621) peak within the 2θ range of 31.4–32° is shown in Figure 1b. Furthermore, the ionic radii of the selected rare-earth element ions are smaller than those of the La ions. As a result, the lattice parameters of META decrease relative to LTA due to the substitution of La with the selected rare-earth element ions. In accordance with Bragg’s law [30], the reduction in lattice parameters results in a shift of the diffraction peak to higher angles. As a result, in comparison to the standard PDF card for LTA, META exhibits a slight shift in the peak position, towards higher angles.

**Figure 2:**
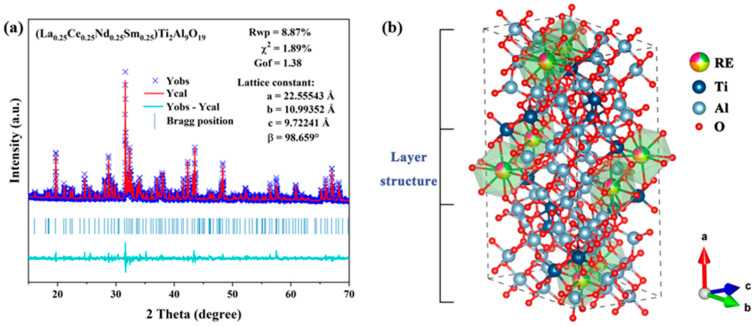
(**a**) Rietveld refinement of XRD patterns of META ceramic powder; (**b**) schematic of the crystal structure of META.

The detailed structure of META was subjected to Rietveld refinement using GSAS-II software, version number 7493c2. The resulting refinement data and the results of the lattice constants of META are presented in Figure 2. The χ^2^ value is less than 3%, the R_wp_ value is less than 10%, and the goodness-of-fit value is between 1 and 2, indicating that the refinement results are reliable. Based on the aforementioned lattice constants, the theoretical density of META can be calculated as 4.381 g·cm*^−^*^3^. The mean value of the actual density of the META bulk samples is 4.021 g·cm*^−^*^3^, which is determined using the Archimedes drainage method. Consequently, the relative density of the META blocks is determined to be 91.78%.

**Figure 3:**
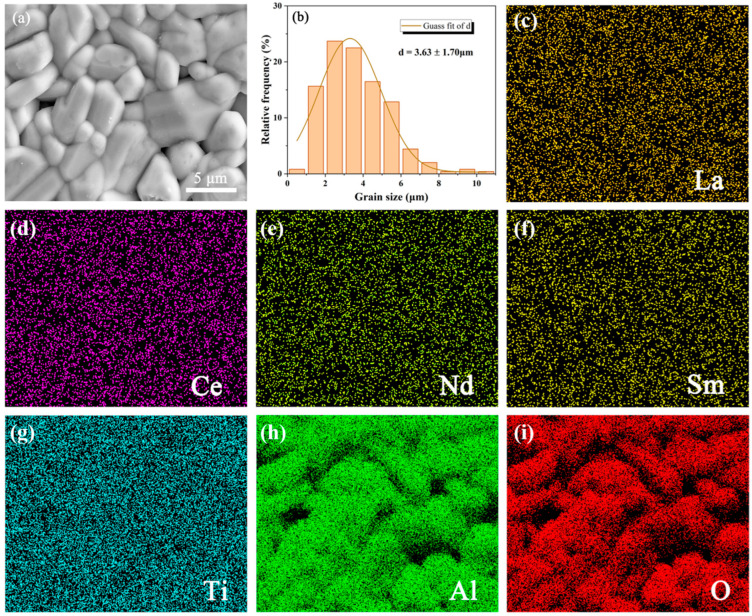
(**a**) Microstructure of the surface; (**b**) grain size distribution; and (**c**–**i**) elements distribution in sintered META ceramic.

The microstructure and element distribution of the META bulk are presented in Figure 3. It demonstrates that the META ceramic displays a relatively dense morphology with a minor degree of porosity and devoid of any visible cracks or other microdefects, which is in accordance with the previously established 91.78% relative density value. The microstructure depicted in Figure 3a exhibits distinct grain boundaries and grains with a consistent size. The grain size distribution presented in Figure 3b was derived from SEM images of varying locations on the surface of the bulk. It suggests that the grain size of META is 3.63 ± 1.70 µm. The majority of the grains of META display a plate morphology. This grain growth behavior can be ascribed to the layered crystal structure observed in Figure 2b. In the layered lattice structure, ion diffusion is slower in the direction perpendicular to the layer plane, but occurs more readily in the direction parallel to it, thereby promoting grain growth in the latter direction [31]. No other products at the grain boundaries can be observed. The elemental distribution analysis in Figure 3c indicates that the rare-earth elements are uniformly distributed throughout the ceramic, with no observable segregation. This homogenous distribution suggests that the chemical composition of the META ceramic is uniform, and that the rare-earth elements have been uniformly incorporated into the lattice structure. The result of elemental distribution analysis is subject to partial influence from the topographic lining due to the substantial O and Al content, resulting in perceptually uneven elemental distribution.

### 3.2. High-Temperature Stability and Thermophysical Properties Characterization

**Figure 4:**
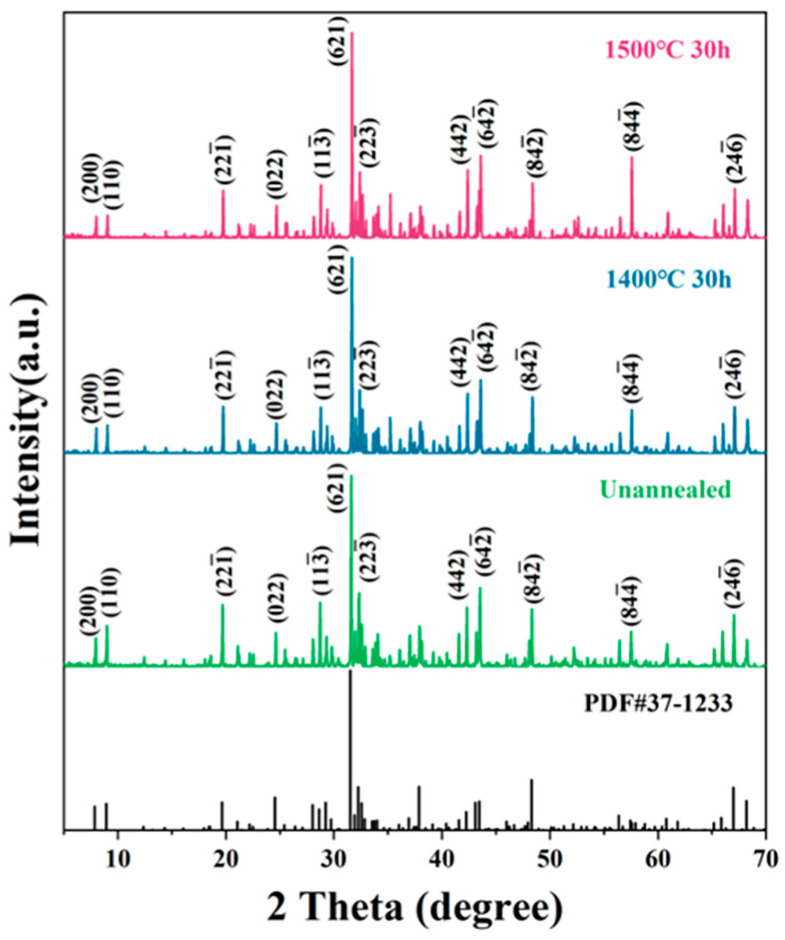
XRD patterns of META and after heating at 1400 °C and 1500 °C for 30 h.

To investigate the thermal stability of META ceramics, pure META powder was synthesized and consolidated into green bodies, which were then subjected to heating treatments at various temperatures. Figure 4 shows the XRD patterns of the META powder before and after heating for 30 h. The XRD patterns indicate the absence of any additional phases or products, thereby confirming the persistence of a single-phase constitution in the post-heat-treatment state.

Furthermore, there is no significant decrease in the relative intensities of any diffraction peaks, indicating that the crystallinity of META ceramics remained consistent under the test conditions. Notably, the peak intensity of the (8,4,$\bar{4}$) plane increases significantly at 1500 °C compared to other temperatures, likely due to grain growth along the optimal crystallographic orientation, rather than changes in stability. Based on these observations, it can be concluded that META exhibits high thermal stability, and that no phase change of the matrix will occur during subsequent corrosion experiments.

**Figure 5:**
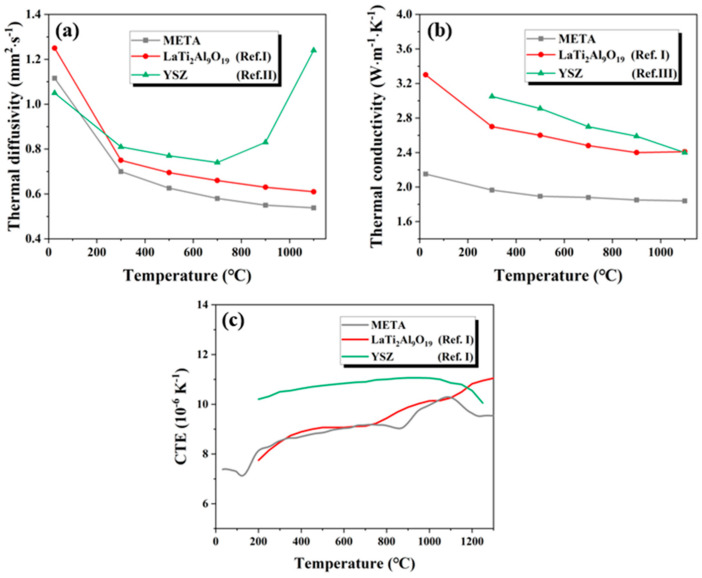
(**a**) The thermal diffusivity; (**b**) thermal conductivity; and (**c**) CTE of META, single-component LTA and YSZ under different temperature. Ref. I [17], Ref. II [32], Ref. III [33].

The thermal conductivity is a pivotal parameter that exerts a considerable influence on the thermal shielding and durability of TBCs. Figure 5 presents a comparison of the thermal diffusivity and thermal conductivity of META with those of the other TBCs’ materials [17,32,33]. Figure 5a,b show that META has lower thermal diffusivity and conductivity than YSZ (0.75–1.25 mm^2^·s^−1^, 2.4–3.1 W·(m·K)^−1^, 25–1100 °C). The thermal diffusivity of META is 1.16 mm^2^·s^−1^ at 25 °C and decreases to 0.58 mm^2^·s^−1^ at 1100 °C, and the thermal conductivity is 2.15 W·(m·K)^−1^ at 25 °C and decreases to 1.84 W·(m·K)^−1^ at 1100 °C. Additionally, the thermal conductivity of META is lower than that of single-component LTA (2.4–3.4 W·(m·K)^−1^, 25–1100 °C), demonstrating its superior thermal barrier performance at elevated temperatures. The thermal conductivity of META exhibits a decline with rising temperatures, suggesting its suitability for high-temperature applications. The synergy effect of the ionic radius and mass differences between doped rare-earth ions induces drastic lattice distortions, which reduces the phonon-effective relaxation time and enhances phonon–phonon scattering [34]. As Equation (7) shows, this effect can be quantitatively evaluated by the phonon scattering coefficient (*Γ*) expression of ceramics materials derived from Callaway and Klemens [35] when the Umklapp phonon–phonon scattering process is followed.

(7) $$\Gamma =f\times {\left[{\left(\frac{\Delta M}{M}\right)}^{2}+2\left[6.4\times \frac{1}{3}\times \gamma \times \frac{1+\sigma }{1-\sigma }\left(\frac{\Delta \delta }{\delta }\right)\right]\right]}^{2}$$

where *f*, Δ*M*, *γ*, Δ*δ*, and *σ* are the defect concentration, mass difference of rare-earth ions, Greenelson constant, ionic radius difference, and Poisson’s ratio, respectively. It can be observed that the incorporation of rare-earth ions with large radii and mass differences into identical lattice positions resulted in an increase in the phonon scattering coefficient, which turns into enhanced phonon–phonon scattering and reduces the thermal conductivity of the material. In conclusion, the low thermal conductivity of META can be attributed to differences in atomic mass, atomic radius mismatch, and fluctuations in the chemical bonds involved by doped rare-earth ions.

The CTE provides insight into the ability of TBCs to accommodate the underlying bonding layer during thermal cycling. Consequently, the thermal expansion behavior of META was investigated. The linear thermal expansion characteristics of META can be described by the following equation, which has been derived through linear fitting to the experimental data:

(8) $$\Delta L/L_{0}=9.1\times 10^{-4}\;T-0.05$$

Figure 5c illustrates the CTE results for META and other TBC materials between 30 °C and 1300 °C. The CTE curves in Figure 5c were derived through the process of fitting the original data. The CTE of META increases with temperature, reaching a maximum value of 10.5 × 10^−6^ K^−1^. This value is comparable to the CTE of standard YSZ (10.50–11.50 × 10^−6^ K^−1^), thereby reducing the risk of failure due to thermal mismatch stress between underlying layers. This indicates that META has the potential to serve as a promising top coating material in a double-layer TBC system. The CTE of the solid materials is affected by the ionic bond intensity, which can be evaluated using Pauling electronegativity by Equation (9) [36].

(9) $$I_{A-B}=1-e^{-\frac{{\left(x_{A}-x_{B}\right)}^{2}}{4}}$$

Where *I_A−B_* are the bond energy between ions at the A and B site, and *x_A_*, *x_B_* are the electronegativity of ions, respectively. The decrease in ionic bond intensity will lead to the increase in non-harmonic vibration amplitude when the temperature increases, manifesting as the increase in CTE. As the electronegativity of lanthanide ions increases with the atomic number, the electronegativity difference between rare-earth ions and O^2−^ ions decreases, resulting in a decrease in ionic bond intensity. Therefore, META exhibits a higher CTE compared to LTA in some temperature ranges. Nevertheless, the CTE of META displays a precipitous decline at elevated temperatures, which may be attributable to sintering shrinkage [37].

In order to facilitate a more robust and nuanced comparison of the thermophysical properties of META and other TBC materials, the thermal conductivity and CTE of these materials are listed in Table 1.

### 3.3. Mechanical Properties

**Figure 6:**
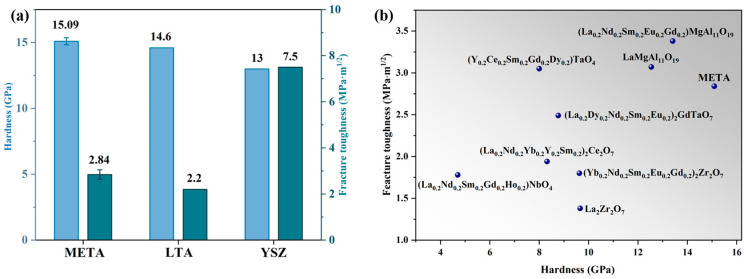
(**a**) Hardness and fracture toughness of META, LTA, and YSZ [17]; (**b**) comprehensive comparison of mechanical properties of META and other materials for TBCs [39,40,49,50,51,52].

The hardness and fracture toughness of META were systematically evaluated and compared to other TBC materials, as illustrated in Figure 6. The incorporation of ions with radii less than La ions has been observed to reduce the average bond length of META, thereby increasing the bond strength and overall structural integrity. Lattice distortions resulting from atomic size mismatch and mass irregularities elevate the internal energy and microscopic stresses, impeding dislocation motion and enhancing the microhardness of META [47]. Consequently, the measured average hardness value of META is 15.09 GPa, which is higher than that of LTA (14.6 GPa) [17]. The enhanced hardness provides increased resistance to particulate erosion and reduces the risk of premature failure as a TBC ceramic top layer due to external physical impacts.

The fracture toughness value was calculated using the equation provided (Equation (5)), and the resulting average value for META is 2.84 MPa·m^1/2^, which is higher than that of LTA (2.20 MPa·m^1/2^). The observed enhancement of fracture toughness can be attributed to the increased lattice distortions and chemical bonding inhomogeneities that result from doping with multiple rare-earth ions. The variability in ionic radii improves structural toughness, and the lattice distortion and structural disorder increase the cohesive energy, which is directly proportional to the fracture energy and is the main determinant of fracture toughness. Furthermore, the tendency of the material to fracture decreases as the cohesive energy increases [48]. Therefore, META exhibits superior fracture toughness compared to single-component LTA. Coupled with its elevated hardness, the fracture toughness of META serves to mitigate thermal mismatch stresses and diminish the probability of coating failure due to thermal cracking.

### 3.4. Infrared Emissivity Properties

**Figure 7:**
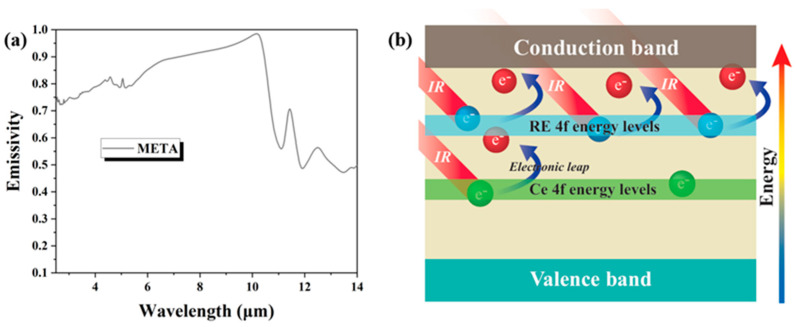
(**a**) Infrared emissivity of META; (**b**) schematics of the possible mechanism for infrared emissivity of META.

The emissivity of META across the wavelength range of 2.5 to 14 μm at 25 °C is presented in Figure 7. The emissivity within the range of a 2.5–6 μm wavelength with a calculated average value of 0.78, and in the range of 2.5–10 μm, is 0.86. The near-infrared radiation becomes predominant at high temperatures [53]. Consequently, the near-infrared emissivity emerges as a pivotal factor influencing the application of high-temperature materials. On the basis of semiconductor theories, the band gap plays a crucial role in electron transitions. The infrared absorption in the range of 2.5–10 μm is related to the energy gap between impurity energy levels and the conduction band. Rare-earth elements, excluding La, contain additional f-electrons, which promote the formation of impurity energy levels near the conduction band, thereby reducing the energy gap between the valence and conduction bands [8]. The presence of additional f-electrons in rare-earth elements results in discrete 4f energy levels near the conduction band, facilitating the absorption of infrared radiation and transitions to higher energy levels [54], and thereby increasing emissivity. The various oxidation states of Ce introduce additional discrete 4f energy levels within the bandgap, further enhancing infrared absorption by f-electrons [55]. The reduction in the energy gap facilitates the transition of surplus f-electrons from impurity energy levels to the conduction band, thus enhancing energy absorption in the range of 2.5–10 μm.

Doping elements also enhance impurity absorption and free carrier absorption within the crystal, further influencing emissivity in the range of 2.5–10 μm. While free carrier absorption is complex and spans a broad wavelength range, its absorption coefficient generally increases with increasing wavelength [56]. Consequently, emissivity rises with an increasing wavelength. According to the semiconductor optical absorption theory, infrared absorption in the range of 10–14 μm is predominantly attributed to lattice vibrations. In this range, significant fluctuations in infrared emissivity are linked to the stretching vibrations of [AlO_4_]^5−^ tetrahedra and [AlO_6_]^9−^ octahedra [57]. The increased atomic disorder in META may lead to changes in electric dipole moment vectors that counteract each other. As the intensity of lattice vibration absorption is influenced by variations in dipole moment vectors, this results in a reduction of absorption intensity in the range of 10–14 μm. Consequently, META exhibits a higher infrared emissivity and absorption, with a reduced photon mean free path due to infrared opacity, thus decreasing the contribution of radiative heat transfer to thermal conductivity and ultimately lowering the thermal conductivity at high temperatures.

### 3.5. CMAS Corrosion Processes

**Figure 8:**
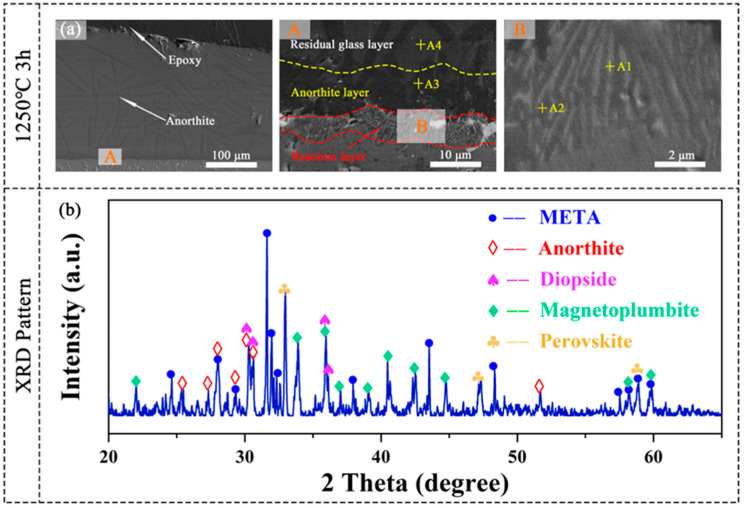
(**a**) Cross-sectional morphology and corresponding high-magnification images (**A**,**B**); (**b**) XRD pattern of META corroded at 1250 °C for 3 h.

**Figure 9:**
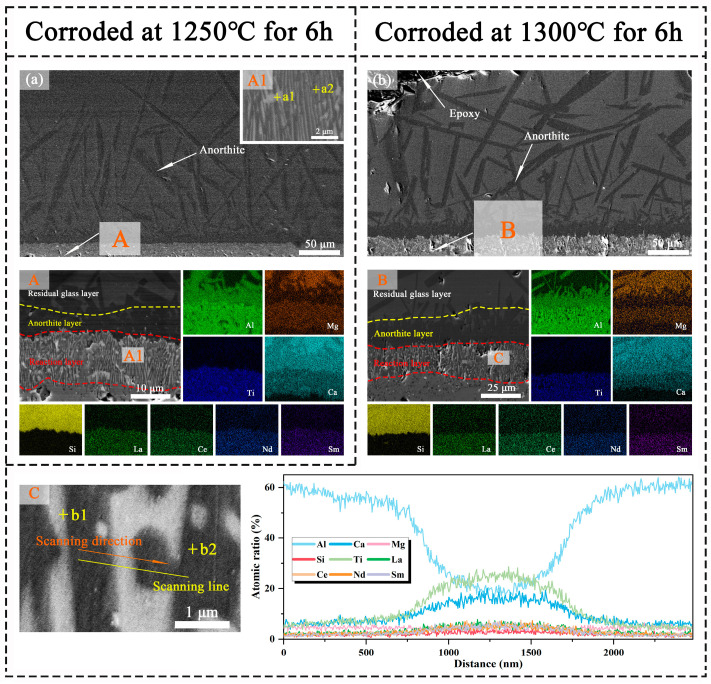
(**a**) Cross-sectional morphology and (**A**,**A1**) corresponding high-magnification images and element distribution of CMAS-corroded META at 1250 °C for 6 h. (**b**) Cross-sectional morphology, (**B**) corresponding high-magnification images, and element distribution. (**C**) Element line scan result of CMAS-corroded META at 1300 °C for 6 h.

**Figure 10:**
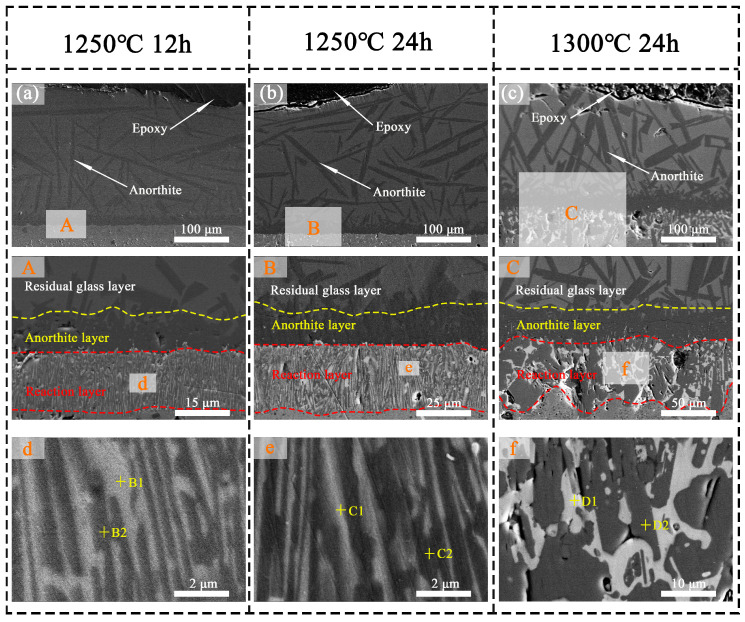
(**a**–**c**) Cross-sectional morphology and (**A**–**C**) corresponding high-magnification images of CMAS-corroded META at 1250 °C for 12 h, 24 h, and at 1300 °C for 24 h; (**d**–**f**) corresponding high-magnification images of regions mentioned in reaction layer.

Corrosion tests were conducted on META at 1250 °C for 3, 6, 12, and 24 h, with additional tests performed at 1300 °C for 6 and 24 h to assess the high-temperature corrosion behavior. The elemental composition and content of the regions identified in Figure 8, Figure 9 and Figure 10 are presented in Table 2 for purposes of summary. Figure 8a depicts the cross-sectional of the META sample that was subjected to molten CMAS corrosion at 1250 °C for 3 h. The results demonstrated that the corrosion interface manifested as a four-layer structure, comprising a light-colored layer at the tip, a plate-like crystal layer, a striated crystal layer, and the matrix. The composition of Region A4 consists primarily of Ca, Mg, Al, Si, and O, and the stoichiometric ratio of Si to Ca is observed to be approximately 1.50, which is comparable with the composition of CMAS and suggests that Region A4 represents the unreacted residual CMAS glass. Region A3 is observed to be uniformly distributed across the section with a dense structure, and is identified as anorthite (CaAl_2_Si_2_O_8_, An), based on the findings of EDS spot-scanning and previous research [58]. Given that anorthite is widely accepted as a CMAS self-crystallization product [59], the striated layer below it can be identified as a reaction layer. The mean thickness of the reaction layer is approximately 5 μm in this section.

In order to ascertain the crystal structure of the products resulting from the corrosion process of CMAS, a mixture of CMAS and META powders in a 10:3 mass ratio was subjected to a heat treatment at 1250 °C for 3 h. Figure 8b illustrates the presence of distinctive peaks for perovskite, magnetoplumbite, diopside, and anorthite in the XRD pattern of the post-treatment sample. In conjunction with the EDS point-scanning results detailed in Table 2, the presence of magnetoplumbite-type hexaaluminate (Mag) and perovskite (Prv) identifies them as corrosion products of META. The extant literature indicates that diopside is a self-crystallization product of CMAS [46]; however, it is not observed in the corrosion interface cross-section. Its presence in the XRD pattern may be attributable to the differences in the contact method and mass ration between CMAS corrosion samples and the XRD sample. The phases corresponding to the indicated points in Figure 8, Figure 9 and Figure 10 are presented in Table 3. For convenience, the names of the corrosion products will be referred to by their abbreviations, as indicated in Table 3.

Figure 9a,b illustrates the cross-sectional microstructure and elemental distribution of META following 6 h of CMAS corrosion at temperatures of 1250 °C and 1300 °C, respectively. The high-magnification images are presented in Figure 9A–C, and the microstructure and elemental distribution of META after 6 h of CMAS corrosion at 1250 °C and 1300 °C are presented with high-magnification images. Following a 6 h period of corrosion, a continuous reaction layer with a dense structure is observed on the surface of the substrate. The mean thickness of the reaction layer is approximately 13 μm, which is observed in the section at 1250 °C. The thickness of the reaction layer exhibited a notable increase following the temperature elevation from 1250 °C to 1300 °C, reaching an average thickness of approximately 23 μm of the section shown in Figure 9B. In order to elucidate the phase composition of the reaction layer, an EDS line scan of the cross-section of the reaction layer is carried out after 6 h of corrosion at 1300 °C. Figure 9C illustrates a discernible variation in the elemental composition of the reaction layer from one layer to the next, which is indicative of the presence of the following two distinct phases: a darker Al-rich phase and a lighter Ti-rich phase. This conclusion is corroborated by the elemental surface scans depicted in Figure 9B. In conjunction with the analysis of the EDS point-scanning results for the points within the reaction layer, as illustrated in Table 2, it can be ascertained that the light-colored streak-like crystals within the reaction layer are of the Prv phase, whereas the Mag phase, which is also a reaction product, is predominantly distributed within the dark-colored region of the reaction layer.

Given that the majority of CMAS corrosion products of META contain Ca, the distribution of this element is employed as a benchmark for the assessment of the penetration of molten CMAS into the ceramic matrix. In order to ascertain whether CMAS had penetrated into the matrix, EDS cross-section scans of the samples were conducted after corrosion at 1250 °C and 1300 °C for 6 h, and are presented in Figure 9A,B. The results demonstrate the existence of a distinct boundary in Ca content between the reaction layer and the matrix, as well as a clear boundary in Si content between the residual glass and the bi-layer. Furthermore, the concentration of rare-earth elements increases from the residual CMAS to the matrix. This indicates that the interior of the ceramic material is not affected by the CMAS molten and that the bi-layer structure comprising An and the reaction layer is effective in preventing the penetration of the molten salts. Furthermore, the EDS line-scanning results indicate a gradual decline in the concentrations of both Ca and rare-earth elements from the Prv-phase region to the Mag-phase region, following a specific gradient. A gradient diffusion of elements between the two phases of the reaction layer is observed. The concentrations of various rare-earth elements exhibit fluctuations in the Mag and Prv regions, indicating that these elements exhibit disparate diffusion rates during the corrosion process, with some elements accumulating in specific regions, as illustrated in Table 2.

Figure 10a,b illustrates the cross-sectional microstructure of META following 12 and 24 h of CMAS corrosion at 1250 °C, while Figure 10c depicts the results after 24 h of corrosion at 1300 °C. The high-magnification images are presented in Figure 10A–C,d–f. Table 2 presents the results of the elemental scanning of the selected points. The morphology of the corroded surfaces at different temperatures exhibits notable variation. Following a prolonged corrosion period at 1250 °C, the composition of the corroded interface remained largely unaltered in comparison to the 3 h and 6 h conditions. The average thickness of the reaction layer at this interface was approximately 19 μm after it was corroded for 12 h. After 24 h, the average thickness of the reaction layer at this cross-section was approximately 28 μm. After 24 h of corrosion at 1300 °C, a continuous CMAS glass residual layer remained above the reaction layer, indicating that the bi-layer system effectively inhibited the penetration of molten salt. In this cross-section, the average thickness of the reaction layer is approximately 59 µm, demonstrating a notable increase. According to the Arrhenius viscosity model (10), the viscosity of high-temperature inorganic melts is usually negatively correlated with temperature.

(10) $$\eta =A\times \exp\left(B/T\right)$$

where *η* and *T* are the viscosity of the melt and experiment temperature, and *A* and *B* are the experimental measurement parameter. Therefore, the permeability of the CMAS melt is enhanced due to the decrease in viscosity, accelerating the formation of the reaction layer. Concurrently, the coarsening and agglomeration of the two phases within the reaction layer can be observed, accompanied by the generation of a certain number of tiny pores. However, these pores did not form significant CMAS infiltration channels due to the obstruction of the An layer situated above the reaction layer. Furthermore, no localized peeling of the reaction layer at 1300 °C, in contrast to what was observed during LTA corrosion, was noted [57].

### 3.6. CMAS Infiltration Inhibiting Mechanism

The CMAS corrosion process primarily involves the interaction between the molten CMAS and META ceramic, resulting in a reaction layer observed to manifest under all corrosion conditions that formed with non-stoichiometric Mag and Prv phases. Based on the discussion above, the chemical equations for the corrosion process can be written as shown in Equation (11).

RETi_2_Al_9_O_19_ + CMAS → (Ca, RE_0.7_)(Ti, Al)O_3_ + RE(Mg, Ca)Al_11_O_19_ + CaAl_2_Si_2_O_8_ (11)

**Figure 11:**
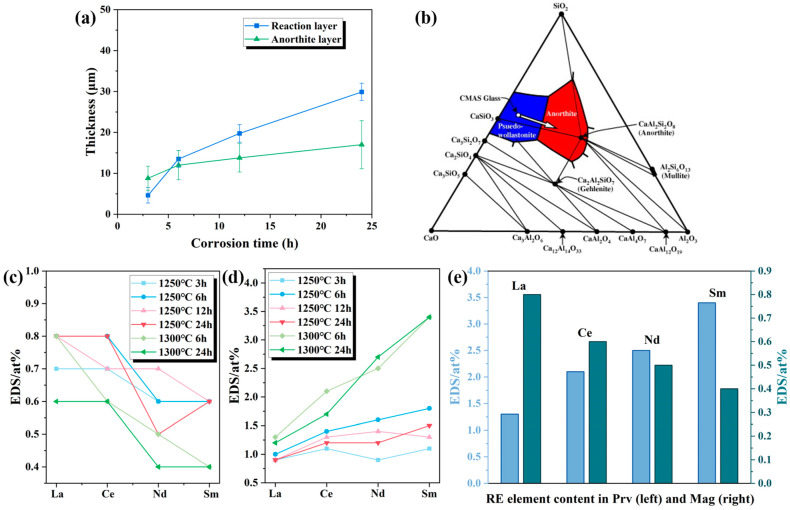
(**a**) The thickness of the reaction and An layers during CMAS corrosion at 1250 °C, (**b**) the CaO-Al_2_O_3_-SiO_2_ (wt.%) ternary phase diagram [67], the content of rare-earth elements in (**c**) the Mag and (**d**) Prv phase zones, (**e**) the content of rare-earth elements in the reaction layer during CMAS corrosion at 1300 °C for 6 h.

Figure 11a illustrates the progressive increase in the thickness of both the An layer and the reaction layer as a function of corrosion time at 1250 °C. As the corrosion time exceeds 6 h, the thickness of the An layer has been maintained at a relatively low increase rate, and has undergone gradual densification over time. Concurrently, the rate of the reaction layer thickness growth is observed to decrease significantly and become more stable over 6 h of corrosion. The CMAS corrosion process is characterized by a dissolution and re-precipitation mechanism [60], whereby molten CMAS infiltrates through the pores and dissolves META particles at the corrosion interface. The An layer is a self-crystallization product and the higher melting point of anorthite renders it relatively insensitive to temperature variations. This finding indicates that the continuous An layer effectively inhibits the dissolution of the matrix into the molten CMAS. Additionally, the An phase has the potential to mitigate corrosion damage to a certain extent due to its ability to increase the melting point of CMAS [15]. Consequently, the bi-layer structure comprising the An layer and the reaction layer plays a pivotal role in preventing further penetration.

Furthermore, the reduced CMAS penetration depth of META in comparison to conventional TBCs’ material can also be ascribed to the increased viscosity of CMAS. The EDS results in Figure 9 and Table 2 indicate that the residual CMAS layer contains a certain stoichiometric ratio of Ti and Al, which, when combined, increase the viscosity of the CMAS melt and reduce the penetration into the matrix. The addition of Ti results in a more extensive spread of molten CMAS on the matrix surface than Ti-free CMAS due to reduced viscosity [61]. Nevertheless, because the basic oxides reduce the viscosity of the glass melt by forming non-bridging oxygen bonds, the viscosity and melting point of CMAS increase when the dissolved Ti ions’ content is sufficient for the consumption of basic oxides in CaO, resulting in the production of CaTiO_3_ and significant amounts of An [62,63,64], both of which can be observed at the corrosion interface. Furthermore, META is capable of dissolving a sufficient quantity of Al during its interaction with CMAS, thereby increasing the relative Al content of CMAS. According to a study by ZHEN [65], the replacement of TiO_2_ in the melt by Al_2_O_3_ results in a reduction in the amount of non-bridging oxygen, which, in turn, causes an increase in viscosity. The rise in viscosity impedes the rate of diffusion of the melt across the ceramic surface, reducing the wettability of the molten CMAS. Concurrently, the degree of penetration through the pores and grain boundaries is markedly diminished when the viscosity of the CMAS increases.

The EDS line scan results presented in Figure 9, in conjunction with the elemental distribution curves illustrated in Figure 11c–e, demonstrate the existence of an elemental gradient diffusion phenomenon between the two phases within the reaction layer. This crystallization rate gradient serves to reduce the rate of formation of the reaction layer, thereby mitigating the thermal stresses caused by the disparate thermal expansion rates of Mag and Prv. This effect serves to inhibit cracking and reduce CMAS infiltration channels [66]. Furthermore, the increasing of a finer and dense microstructure within the reaction layer enhances the failure tolerance of the reaction layer under performance conditions. Due to the relatively high configuration entropy, META exhibits robust high-temperature chemical stability and a relatively slow dissolution rate in the melt, and residual CMAS residues are observed after 24 h of corrosion. As a result, the reaction layer of META is thin in comparison to that of one-component LTA, demonstrating superior resistance to molten salt corrosion.

As demonstrated in Figure 11c–e, the distribution of rare-earth elements in the two phases within the reaction layer system exhibits a discernible size tendency. This tendency is characterized by the enrichment of rare-earth elements with larger radii in the Mag phase, while the Prv phase demonstrates comparatively higher content of small-radius rare-earth ions. This trend becomes more pronounced at higher corrosion temperatures. This phenomenon may attribute to fitness differences between ion size and crystal structure due to compactness of lattice structure, which can be described by the ionic packing factor. For a compound (AxByCz), it can be determined using the following Equation (12) [67]:

(12) $$Ionic\;packing\;factor=Z\;\left(xV_{A}+yV_{B}+zV_{C}\right)÷V_{cell}$$

where $xV_{A}$, $yV_{B}$, and $zV_{C}$ are ion volumes calculated based on the ionic radii, Z is the number of the formula in a unit cell, and $V_{cell}$ is the unit cell volume. The lower ionic packing factor offered by the magnetoplumbite lattice provides diffusion channels and a low-strain environment for large ions, allowing the large rare-earth ions to diffuse into and remain in the Mag phase. In addition, in contrast to the corrosion behavior exhibited by LTA, the reaction layer of META remains intact with the matrix, and no peeling of the reaction layer occurs as the temperature is increased to 1300 °C [68]. According to Figure 11c–e, there is still a considerable amount of small-sized rare-earth ions distributed in the reaction layer. Based on the Born Landé theory, the lattice energy (*U*) is related to the ionic radius in ionic compounds, and it can be approximated by Equation (13).

(13) $$U=\frac{N_{0}AZ^{+}Z^{-}e^{2}}{4\pi \varepsilon_{0}r_{0}}\;\left(1-\frac{1}{n}\right)$$

where, *n*, *e*, A, $Z^{+}$, $Z^{-}$, $N_{0}$, $\varepsilon_{0}$, and $r_{0}$ are the Born exponent, electron charge, Madelung constant, positive ionic charge, negative ionic charge, Avogadro constant, permittivity of free space, and interionic distance, respectively. In general, the lattice energy increases with the decrease in interionic distance. For most inorganic materials, the crystallization temperature ($T_{m}$) is proportional to lattice energy (*U*) and can be fitted with the empirical relation $T_{m}=kU$. Therefore, the introduction of small-radius rare-earth elements serves to enhance the lattice energy of the reactive layer, and leads to an improvement in both its crystallization temperature and thermal stability. Concurrently, the aforementioned result offers a thermodynamic rationale for the resilience of META against penetration of the CMAS melt. The risk of decomposition and softening of the reaction layer at a high temperature is reduced, to a certain extent. The formation of permeation channels due to dissolution or a phase change of the reaction layer is avoided and prevents the stripping of the reaction layer of META at 1300 °C, which is markedly distinct from that observed in LTA.

**Figure 12:**
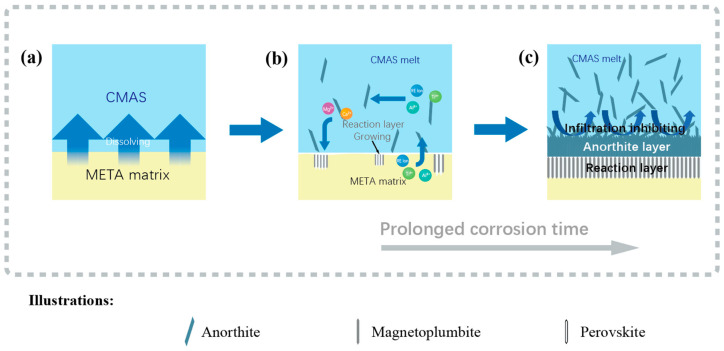
Schematics of CMAS corrosion processes and mechanism on META surfaces at 1250 °C and 1300 °C. (**a**) The temperature has reached the CMAS melting point; (**b**) The bi-layer structure forming at the corrosion interface; (**c**) The observed morphology of the corrosion interface.

Figure 12 provides the schematic representation of the CMAS corrosion mechanism observed in the META samples. In the case of bulk samples, corrosion predominantly follows a thermodynamic trajectory. Initially, the CMAS melts entirely and disseminates to the surface of the mid-entropy bulk sample when the temperature surpasses the melting point of the CMAS. Given that META is an Al-rich and Al-based oxide, it can be observed that the Al content of molten CMAS that deposits in close proximity to META increases, which is in-line with the dissolution process of META through the dissolution and re-precipitation mechanism. As illustrated in the CaO-Al_2_O_3_-SiO_2_ ternary phase diagram shown in Figure 11b [68], the elevated Al concentration in the glassy phase prompted a shift in the CMAS composition from the pseudo-wollastonite field to the anorthite field, as a consequence of the elevated Al concentration. In addition to this, TiO_2_ is a frequently utilized nucleating agent in glass crystallization [67]. Therefore, the transition of the CMAS glass composition towards the anorthosite domain, coupled with the presence of TiO_2_ nucleating agents within this glass, has resulted in the crystallization of the CMAS glass front into crystalline anorthite. As a result, an An layer is observed to crystallize at the surface of the META sample. Additionally, there are relevant research findings which show that the solvation of rare-earth elements in CMAS creates favorable conditions for the nucleation of the An phase [69].

## 4. Conclusions

In this study, a new type of mid-entropy rare-earth aluminate ceramic was designed and successfully synthesized through the solid-state reaction method. Additionally, its microstructure and compositions, as well as its mechanical, thermophysical, thermo-optical, and CMAS corrosion resistance properties, were systematically examined. The main conclusions are as follows:

(1) META exhibits exceptional thermophysical and mechanical properties. The incorporation of atoms with different radii results in low thermal conductivity (1.84–2.15 W·(m·K)^−1^). The experimental results demonstrated that the CTE of META exhibits fluctuations with temperature, which has an impact on its thermal cycling performance. However, in general, the CTE of META is comparable to that of YSZ, which significantly mitigates the detrimental effects of thermal mismatch on its thermal shielding performance.
(2) META has a high hardness (15.10 GPa), good fracture toughness (2.20 MPa·m^1/2^), and high infrared emissivity in the range of 2.5 to 10 μm, with an average value of 0.86. The mechanical properties of META can assist in the prolongation of the operational lifespan of TBCs. Consequently, at elevated temperatures META becomes infrared opaque, reducing the photon mean free path and minimizing the contribution of radiative heat transfer to thermal conductivity. This ultimately reduces the thermal conductivity at high temperatures.
(3) META has been demonstrated to be an effective inhibitor of corrosion in CMAS. The combined effects of delayed diffusion, increased viscosity of molten CMAS, the barrier effect of the self-crystallized product layer, and improved high-temperature stability of the reaction layer serve to enhance the ability of META to resist the penetration and corrosion of molten CMAS.

## Figures and Tables

**Table 1:** Comparison of the thermophysical properties of META to other TBCs.

Compounds	Thermal Conductivity (W·(m·K)^−1^)	CTE (10^−6^ K^−1^)
META (This work)	1.84–2.15, 300–1100 °C	8.13–10.50, 200–1300 °C
YSZ [38]	2.40–3.04, 100–900 °C	11, 1200 °C
LaMgAl_11_O_19_ [39]	2.62, 1000 °C	8.53, 1300 °C
(La_0.2_Nd_0.2_Sm_0.2_Eu_0.2_Gd_0.2_)MgAl_11_O_19_ [39]	2.19–3.27, 25–1000 °C	9.22, 1300 °C
La_2_Zr_2_O_7_ [40]	2.3, 1000 °C	9.1, 1200 °C
(Dy_0.2_Y_0.2_Ho_0.2_Er_0.2_Yb_0.2_)_2_Zr_2_O_7_ [40]	2.18, 1000 °C	10.08, 1200 °C
YTaO_4_ [41]	1.57–2.88, 100–800 °C	9.6, 1200 °C
GdTaO_4_ [41]	1.83–3.61, 100–800 °C	8.8, 1200 °C
(Nd_0.25_Sm_0.25_Eu_0.25_Gd_0.25_)TaO_4_ [42]	2.1–2.97, 100–1000 °C	8.8, 1200 °C
(Nd_0.2_Sm_0.2_Eu_0.2_Gd_0.2_Dy_0.2_)TaO_4_ [42]	1.94–3.13, 100–1000 °C	9, 1200 °C
La_2_Hf_2_O_7_ [43]	1.44, 1200 °C	9.2, 1300 °C
(La_0.2_Ce_0.2_Pr_0.2_Sm_0.2_Eu_0.2_)_2_Hf_2_O_7_ [44]	1.00, 800 °C	12.7, 1200 °C
(Dy_0.25_Er_0.25_Y_0.25_Yb_0.25_)_3_NbO_7_ [45]	0.9, 25 °C	9.8, 1200 °C
(Y_0.25_Ho_0.25_Er_0.25_Yb_0.25_)_2_SiO_5_ [46]	1.0–1.47, 200–800 °C	6.6, 1460 °C

**Table 2:** The element content (at%) of marked points in Figure 8, Figure 9 and Figure 10.

Corrosion Condition	Position/at%	Ca	Si	Mg	La	Ce	Nd	Sm	RE Total	Ti	Al	O
1250 °C/3 h	A1	7.2	-	1.5	0.9	1.1	0.9	1.1	4.0	9.2	23.7	54.4
	A2	4.4	-	2.3	0.7	0.7	0.6	0.6	2.6	4.3	35.6	50.9
	A3	12.9	27.5	-	-	-	-	-	-	-	23.4	35.8
	A4	19.7	29.7	6.1	0.2	0.3	0.3	0.3	1.1	1.4	5.4	36.3
1250 °C/6 h	a1	9.9	-	1.1	1.0	1.4	1.6	1.8	4.8	14.1	18.5	50.5
	a2	4.2	-	2.2	0.8	0.8	0.6	0.6	2.8	4.2	37.3	49.4
1250 °C/12 h	B1	12.2	-	1.3	0.9	1.3	1.4	1.3	4.9	13.9	15.7	51.9
	B2	3.8	-	2.3	0.8	0.7	0.7	0.6	2.8	4.1	37.9	50.4
1250 °C/24 h	C1	12.1	-	1.4	0.9	1.2	1.2	1.5	4.8	14.5	14.8	51.3
	C2	3.6	-	2.2	0.8	0.8	0.5	0.6	2.7	3.4	39.9	50.7
1300 °C/6 h	b1	10.9	-	-	1.3	2.1	2.5	3.4	9.3	19.7	9.5	50.2
	b2	2.5	-	2.7	0.8	0.6	0.5	0.4	2.3	1.6	41.5	48.0
1300 °C/24 h	D1	12.6	-	-	1.2	1.7	2.7	3.4	9.0	21.9	3.5	52.7
	D2	2.0	-	2.6	0.6	0.6	0.4	0.4	2.0	1.2	42.86	49.6

**Table 3:** The phase corresponding to the marked points in Figure 8A,B, Figure 9A1,C and Figure 10d–f.

Chemical Formula	Crystalline Structure	Abbreviation	Marked Points	Description
CaAl_2_Si_2_O_8_	Anorthite	An	A3	Calcium aluminosilicate
RE(Ca, Mg)Al_11_O_19_	Magnetoplumbite	Mag	a2, b2, A2, B2, C2, D2	Magnetoplumbite-type rare-earth hexaaluminate
(Ca, RE)(Ti, Al)O_3_	Perovskite	Prv	a1, b1, A1, B1, C1, D1	Multicomponent perovskite solid solution
Ca_33_Mg_9_Al_13_Si_45_	-	CMAS	A4	Molten salts

## Data Availability

The original contributions presented in this study are included in the article. Further inquiries can be directed to the corresponding author.
